# Identification and Quantification of Iron Metabolism Landscape on Therapy and Prognosis in Bladder Cancer

**DOI:** 10.3389/fcell.2022.810272

**Published:** 2022-02-21

**Authors:** Xiaodong Song, Sheng Xin, Yucong Zhang, Jiaquan Mao, Chen Duan, Kai Cui, Liang Chen, Fan Li, Zheng Liu, Tao Wang, Jihong Liu, Xiaming Liu, Wen Song

**Affiliations:** ^1^ Department of Urology, Tongji Hospital, Tongji Medical College, Huazhong University of Science and Technology, Wuhan, China; ^2^ Department of Geriatric, Tongji Hospital, Tongji Medical College, Huazhong University of Science and Technology, Wuhan, China

**Keywords:** bladder cancer, iron metabolism, tumor microenvionment, prognostic signature, nomogram, bioinformactics

## Abstract

The morbidity of bladder cancer (BLCA) is high and has gradually elevated in recent years. BLCA is also characterized by high recurrence and high invasiveness. Due to the drug resistance and lack of effective prognostic indicators, the prognosis of patients with BLCA is greatly affected. Iron metabolism is considered to be a pivot of tumor occurrence, progression, and tumor microenvironment (TME) in tumors, but there is little research in BLCA. Herein, we used univariate COX regression analysis to screen 95 prognosis-related iron metabolism-related genes (IMRGs) according to transcription RNA sequencing and prognosis information of the Cancer Genome Atlas (TCGA) database. TCGA-BLCA cohort was clustered into four distinct iron metabolism patterns (C1, C2, C3, and C4) by the non-negative matrix factorization (NMF) algorithm. Survival analysis showed that C1 and C3 patterns had a better prognosis. Gene set variant analysis (GSVA) revealed that C2 and C4 patterns were mostly enriched in carcinogenic and immune activation pathways. ESTIMATE and single sample gene set enrichment analysis (ssGSEA) also confirmed the level of immune cell infiltration in C2 and C4 patterns was significantly elevated. Moreover, the immune checkpoint genes in C2 and C4 patterns were observably overexpressed. Studies on somatic mutations showed that the tumor mutation burden (TMB) of C1 and C4 patterns was the lowest. Chemotherapy response assessment revealed that C2 pattern was the most sensitive to chemotherapy, while C3 pattern was the most insensitive. Then we established the IMRG prognosis signature (IMRGscore) by the least absolute shrinkage and selection operator (LASSO), including 13 IMRGs (TCIRG1, CTSE, ATP6V0A1, CYP2C8, RNF19A, CYP4Z1, YPEL5, PLOD1, BMP6, CAST, SCD, IFNG, and ASIC3). We confirmed IMRGscore could be utilized as an independent prognostic indicator. Therefore, validation and quantification of iron metabolism landscapes will help us comprehend the formation of the BLCA immunosuppressive microenvironment, guide the selection of chemotherapeutic drugs and immunotherapy, and predict the prognosis of patients.

## Introduction

Bladder cancer (BLCA) is one of the most familiar malignant tumors in the urinary system, with about 81400 new cases and 17900 deaths in the United States in 2020 (Siegel et al., 2020). Approximately 75% of BLCA was found to be non-muscle invasive bladder cancer (NMIBC), which was characterized by a high recurrence rate (45% 5-year recurrence rate) (Berdik, 2017; Babjuk et al., 2019). Transurethral resection of bladder tumor (TURBT), chemotherapy, BCG vaccine, radiotherapy, and radical cystectomy are the main treatments for BLCA patients (Berdik, 2017). Chemotherapy and immunotherapy are also important strategies for conservative treatment of BLCA (Yin et al., 2016; Rouanne et al., 2018). However, some patients are not sensitive to these drug therapies. Due to the high recurrence rate, high metastatic risk, and patient’s dissatisfaction with the treatment effect, it is of great significance to identify and quantify some molecular landscapes that have impacts on the choice of drug treatment, and explore a novel indicator that predicts the prognosis of BLCA patients.

Iron is a vital trace element for cell proliferation and growth in the human body (Torti and Torti, 2013). In cancer, the absorbability, effusion, storage, and regulation of iron are entirely disturbed, which indicates that the reprogramming of iron metabolism would induce the dysregulation in tumor cells division and survival (Andrews, 2008; Manz et al., 2016; Wang et al., 2018; Jung et al., 2019). Iron plays a dual role in cancer (Thévenod, 2018). Epidemiological investigations revealed that excess iron was a hazard factor of carcinogenesis (Stevens et al., 1994; Wu et al., 2004; Fonseca-Nunes et al., 2014). The accumulation of iron supports tumor worsening in proliferation, metabolism, and metastasis (Torti et al., 2018). Cancer cells exhibit a phenotype search for iron through disordering regulation of iron-binding proteins (Dufès et al., 2013; Bialasek et al., 2019). On the other hand, iron dependence of cancer cells affects many cell death modes, including ferroptosis, a form of iron-dependent cell death (Mou et al., 2019; Battaglia et al., 2020). Inducing ferroptosis of cancer cells has become a new hotspot in the research and development of cancer treatment (Hassannia et al., 2019; Xu et al., 2019).

There has been little research on iron metabolism in BLCA. The study was conducted to confirm whether iron metabolism had an effect on the molecular microenvironment of BLCA, as well as its ability to predict the clinical prognosis. We first clustered the TCGA-BLCA cohort into different iron metabolic patterns on the basis of the expression of iron metabolism-related genes (IMRGs). Then the survival prognosis, GSVA analysis, tumor immune microenvironment (TIME), somatic mutations chemotherapy, and immunotherapy response among different patterns were analyzed. Eventually, we established a prognostic signature associated with iron metabolism and confirmed that it is an effective independent predictor in BLCA patients.

## Materials and Methods

### Retrieval of Iron Metabolism-Related Genes

A set of IMRGs was sorted from multiple gene sets from Molecular Signatures Database (MSigDB) (http://www.gsea-msigdb.org/gsea/msigdb/index.jsp), including GOMF_IRON_ION_BINDING, GOBP_IRON_ION_TRANSPORT, GOBP_RESPONSE_TO_IRON_ION, GOBP_IRON_ION_METABOLISM, GOBP_IRON_IMPORT_INTO_CELL, GOBP_IRON_ION_IMPORT_ACROSS_PLASMA_MEMBRANE, GOMF_2_IRON_2_SULFUR_CLUSTER_BINDING, GOMF_4_IRON_4_SULFUR_CLUSTER_BINDING, GOBP_IRON_COORDINATION_ENTITY_TRANSPORT, GOBP_CELLULAR_IRON_ION_METABOLISM, GOBP_HEME_METABOLIC_PROCESS, HEME_BIOSYNTHETIC_PROCESS, MODULE_540, HALLMARK_HEME_METABOLISM and REACTOME_IRON_UPTAKE_AND_TRANSPORT. After removing the duplicate genes from all gene sets, a total of 515 IMRGs were retrieved.

### Acquisition and Process of Original Data

Transcription RNA sequencing, clinical data, and somatic mutation data for patients with BLCA were obtained from the Cancer Genome Atlas (TCGA) database (https://portal.gdc.cancer.gov/). The cohort included 411 BLCA tissues and 19 normal bladder tissues. The TCGA-BLCA level 3 RNA-sequencing data was downloaded as fragments per kilobase of transcript per million mapped reads (FPKM), and when multiple Ensembl IDs were mapped to a single gene symbol in the RNA sequencing data, gene expression is annotated in an average expression. The GSE13507 dataset was analyzed as an external validation cohort. The gene expression profile and clinical information for the microarray dataset came from the Gene Expression Synthesis (GEO) database (https://www.ncbi.nlm.nih.gov/geo/). All sequencing data were processed with log two transformation, background adjustment, normalization, final summarization through the “Affy” package in R. The clinical information of all BLCA patients included in this study is shown in Tables 1, 2.

**TABLE 1:** Characteristics of patients included in the study.

Variable	TCGA-BLCA cohort (n = 400)	GSE13507 cohort (n = 165)
	Number (%)	Number (%)
Age		
≤70	228 (57.00)	109 (66.06)
>70	172 (43.00)	56 (33.94)
Gender		
MALE	296 (74.00)	135 (81.82)
FEMALE	104 (26.00)	30 (18.18)
T stage		
TX	1 (0.25)	0
T0	1 (0.25)	0
Ta	0	24 (14.55)
T1	3 (0.75)	80 (48.48)
T2	117 (29.25)	31 (18.79)
T3	190 (47.5)	19 (11.52)
T4	57 (14.25)	11 (6.67)
Unknow	31 (7.75)	0
N stage		
NX	36 (9.00)	1 (0.61)
N0	233 (58.25)	149 (90.30)
N1	44 (11.00)	8 (4.85)
N2	75 (18.75)	6 (3.64)
N3	7 (1.75)	1 (0.61)
Unknow	5 (1.25)	0
M stage		
MX	194 (48.50)	0
M0	193 (48.25)	158 (95.76)
M1	11 (2.75)	7 (4.24)
Unknow	2 (0.50)	0
Pathologic stage		
Stage 0	0	23 (13.94)
Stage I	2 (0.50)	80 (48.48)
Stage II	127 (31.75)	26 (15.76)
Stage III	138 (34.50)	29 (17.58)
Stage IV	131 (32.75)	7 (4.24)
unknow	2 (0.50)	0
Histologic grade		
Low grade	20 (5.00)	105 (63.64)
High grade	377 (94.25)	60 (36.36)
unknow	3 (0.75)	0

**TABLE 2:** Prognosis-related IMRGs selected by univariate COX regression analysis.

Gene	HR	z	*p*-value
AIFM3	0.73913	−3.0568	0.00224
ALKBH2	0.71241	−2.4545	0.01411
ALKBH3	1.35869	2.1987	0.02790
ALOX5	0.85793	−2.8965	0.00377
ASIC3	0.68739	−2.2624	0.02367
ATP5IF1	0.71955	−2.1818	0.02913
ATP6V0A1	1.81787	3.6315	0.00028
ATP6V0D1	1.45896	2.7342	0.00625
ATP6V1A	1.37125	2.0984	0.03587
ATP6V1C2	1.28480	2.2647	0.02353
ATP6V1G3	2.54732	2.9203	0.00350
BMP6	1.30778	2.9345	0.00334
CAST	1.33555	2.8334	0.00460
CDO1	1.25977	2.3200	0.02034
CIAO3	0.64021	−2.3552	0.01851
CIR1	0.52578	−3.1615	0.00157
CISD1	1.28174	2.0195	0.04343
CLTC	1.52718	3.0834	0.00205
CROCCP2	0.66104	−2.9905	0.00278
CTSE	0.84792	−3.9943	0.00006
CUL1	1.46334	2.1223	0.03381
CYBRD1	1.13803	2.1286	0.03329
CYP19A1	1.82637	3.2558	0.00113
CYP1B1	1.12555	2.3874	0.01697
CYP26B1	1.22486	2.8893	0.00386
CYP27B1	0.76528	−2.0634	0.03908
CYP2C8	0.48055	−3.2681	0.00108
CYP2D6	0.53783	−2.5791	0.00991
CYP2D7	0.39076	−3.3122	0.00093
CYP2F1	1.47375	2.3839	0.01713
CYP2R1	0.68076	−2.2516	0.02434
CYP2W1	1.14759	2.2957	0.02169
CYP3A5	0.88535	−2.1530	0.03132
CYP4A22	0.01075	−2.3088	0.02095
CYP4F12	0.85576	−3.1193	0.00181
CYP4F8	0.89174	−2.6547	0.00794
CYP4Z1	0.51083	−3.0875	0.00202
CYP4Z2P	0.71692	−3.1295	0.00175
CYP51A1	1.54962	3.0788	0.00208
CYP7B1	1.25543	2.0346	0.04189
DNM2	0.72877	−2.6658	0.00768
ENDOD1	1.36254	3.4875	0.00049
EPOR	0.79640	−2.2602	0.02381
FA2H	0.86757	−2.3261	0.02001
FECH	1.29593	2.0714	0.03832
FTO	1.44207	2.1268	0.03344
G6PD	1.18156	2.4243	0.01534
GCLM	1.19098	2.5395	0.01110
HJV	0.00736	−2.2164	0.02667
HRG	0.28674	−1.9665	0.04924
IFNG	0.72938	−2.4603	0.01388
ISCU	0.69174	−2.1696	0.03004
LAMP2	1.22151	2.0780	0.03771
MBOAT2	1.31363	3.5807	0.00034
MKRN1	0.66446	−2.3893	0.01688
MYC	1.14183	2.4370	0.01481
NARF	0.73477	−2.3869	0.01699
NDUFV2	0.71090	−2.3388	0.01934
NFE2	1.25892	2.0291	0.04244
NUBPL	1.71372	2.1941	0.02823
OGFOD1	1.56046	2.0597	0.03943
P3H1	1.27676	2.5856	0.00972
P3H3	1.16777	2.4532	0.01416
P4HA2	1.33888	2.9448	0.00323
P4HA3	1.28168	2.8335	0.00460
PGLS	0.75124	−1.9790	0.04782
PHF8	0.73442	−2.3199	0.02034
PLOD1	1.32204	2.9487	0.00319
PPEF1	1.96121	2.6470	0.00812
PTGIS	1.13599	2.9572	0.00310
RAB11B	0.67495	−2.4727	0.01341
RBM5	0.73620	−2.2846	0.02234
REV3L	1.40219	2.3541	0.01857
RNF19A	0.71735	−3.2263	0.00125
RSAD1	0.73571	−2.1465	0.03184
SCD	1.14688	2.6027	0.00925
SIDT2	1.45914	2.4669	0.01363
SLC25A28	0.75484	−2.0647	0.03895
SLC25A38	0.71371	−2.4859	0.01292
SLC39A14	1.24303	2.5771	0.00996
SLC6A9	1.23132	2.3060	0.02111
SLC7A11	1.16394	2.7767	0.00549
SRI	0.78060	−1.9652	0.04939
STEAP4	1.17801	2.3779	0.01741
TCIRG1	0.70379	−4.0200	0.00006
TET1	2.00528	2.6694	0.00760
TFRC	1.16560	2.2608	0.02377
TMCC2	1.38726	2.8966	0.00377
TNS1	1.11924	2.0886	0.03674
TSPO	0.79418	−2.8037	0.00505
TYW5	0.46331	−2.2249	0.02609
UCP2	0.89329	−2.0547	0.03991
UGT1A1	0.76531	−2.2396	0.02512
UGT1A4	6.38284	2.1260	0.03350
YPEL5	0.62817	−3.0093	0.00262

HR, hazard ratio.

### NMF Clustering for Iron Metabolism Patterns

We matched IMRGs’ RNAseq data and overall survival (OS) information of the TCGA-BLCA dataset. A univariate Cox regression analysis was carried out to determine the prognosis-related IMRGs in BLCA, with a screening criterion of *p* < 0.05. Non-negative matrix factorization (NMF) is applied to determine distinct iron metabolism-related patterns with the help of the “NMF” R package. NMF algorithm decomposes the original matrix into two non-negative matrices to identify the potential features in the gene expression profile (Brunet et al., 2004). Repeat the deposition and aggregate the results to obtain consistent clustering. According to the cophenetic coefficient, contour, and sample size, k = 4 was determined as the best cluster number.

### GSVA

Gene set variant analysis (GSVA), is a nonparametric, unsupervised algorithm. GSVA transforms the isolate gene expression matrix to an expression matrix of particular gene sets as features. The algorithm is implemented based on the “GSVA” R package. The difference of expression matrix after transformation was analyzed by the “limma” package to find the difference of enriched functions among different iron metabolic patterns.

### Evaluation of Tumor Immune Microenvironment

In order to assess the TIME status of BLCA, we used single sample gene set enrichment analysis (ssGSEA), ESTIMATE, and CIBERSORT in R. ssGSEA investigated congenital and adaptive immune cells as well as a variety of immune-related functions. The Normalized Enrichment Score (NES) was to embody the relative amount of each TIME infiltration unit in patients. ESTIMATE predicted the level of infiltrating matrix and immune cells by calculating stromal and immune scores, comprehensively obtained the ESTIMATE score for evaluating the TIME. We also evaluated the relative fraction of 22 tumor-infiltrating immune cells (TIICs), including B cells, T cells, natural killer (NK) cells, macrophages, dendritic cells (DCs), eosinophils, neutrophils, and so on in each cancer sample with CIBERSORT algorithm. *p* < .05 was the threshold of a credible sample for estimating the proportion of immune cells.

### Evaluation of Immunotherapy and Chemotherapy on Iron Metabolism Patterns

Tumor Immune Dysfunction and Exclusion (TIDE) score is a computational framework developed based on the analysis and modeling of characteristic genes for T cell exclusion and T cell dysfunction in immunosuppression at high levels of cytotoxic T lymphocytes (CTL) (Jiang et al., 2018). We applied four indicators to predict the efficacy of immunotherapy, including exclusion score, dysfunction score, microsatellite instability (MSI), and TIDE. The chemotherapeutic response of BLCA patients was evaluated by Genomics of Drug Sensitivity in Cancer (GDSC) (https://www.cancerRxgene.org). Six chemotherapeutic drugs in BLCA treatment were chosen, including Gemcitabine, Cisplatin, Docetaxel, Mitomycin-C, Doxorubicin, and Paclitaxel. The ridge regression algorithm was used to calculate the half-maximal inhibitory concentration (IC50), and satisfactory prediction accuracy was obtained through 10 times cross-validation (Geeleher et al., 2014). The calculation process was completed by the “pRRophetic” R package.

### Construction and Validation of IMRG Prognostic Signature

According to the prognosis-related IMRGs in the univariate Cox regression model, the “glmnet” package in R performed the least absolute shrinkage and selection operator (LASSO) to identify important prognostic IMRGs and select one standard error (SE) above the minimum criteria. The multivariate Cox regression model made it more optimized and practical. Finally, the IMRGscore formula was obtained:

$$\begin{array}{c} \mathrm{Risk}\;\mathrm{score}=\left(\exp\;\mathrm{Gene}1\times \mathrm{coefficient}\;\mathrm{gene}1\right)\\ +\left(\exp\;\mathrm{Gene}2\times \mathrm{coefficient}\;\mathrm{gene}2\right)+\ldots \\ +\left(\exp\;\mathrm{GeneN}\times \mathrm{coefficient}\;\mathrm{GeneN}\right) \end{array}$$

On the basis of the optimal cut-off of IMRGscore obtained by the “surv_cutpoint” function in R, we divided BLCA patients into high-risk and low-risk groups. With the help of Kaplan-Meier analysis (“survival” package) and receiver operating characteristic (ROC) curve (“timeROC” package), the predictive ability of the prognostic signature was evaluated. The diagnostic accuracy was estimated by the area under the curve (AUC). The same IMRGscore calculation formula, cut-off value and, analysis methods were applied in the GSE13507 cohort to validate the signature.

### Establishment and Evaluation of the Nomogram

Nomogram is an intuitive clinical prognosis prediction model integrating a variety of clinicopathological features related to prognosis. We established a nomogram model to provide a more accurate prediction of prognosis for clinical patients based on IMRGscore and clinical pathological characteristics. First, univariate Cox regression analysis was utilized to assess the predicted values of variables. Then further determined the coefficient via multivariate Cox regression analysis. The “rms” R package then established a nomogram for predicting the operating system. Concordance index (C-index) and calibration analysis were applied to estimate the accuracy and consistency. Finally, the clinical application value of the nomogram is evaluated using Decision Curve Analysis (DCA). These analyses were performed with “survival” package.

### Statistical Analysis

All statistical analyses were completed with R software (version 4.0.4) in this study. Before establishing and verifying the prognostic signature, the batch differences between the TCGA dataset and GEO datasets were removed through the “sva” package. Wilcoxon rank-sum test was to verify the significance of the difference in two groups. When comparing more than two groups, the Kruskal Wallis test was selected to verify the difference. Set *p*-value <.05 as a statistically significant standard.

## Results

**FIGURE 1:**
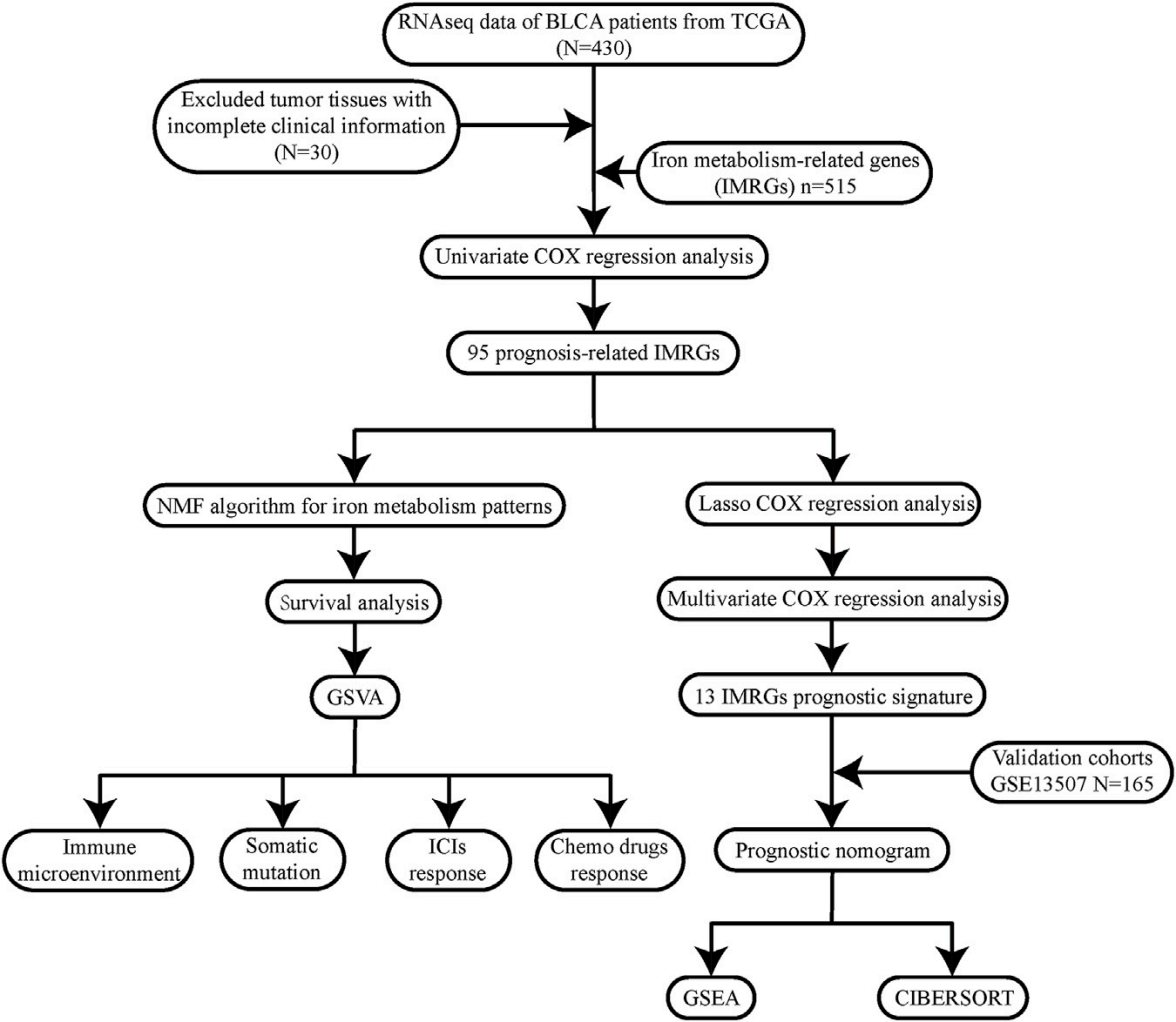
Flow chart of our study.

To describe our research intuitively and systematically, we showed the research process in Figure 1.

### Characterization of Iron Metabolism Patterns in BLCA

**FIGURE 2:**
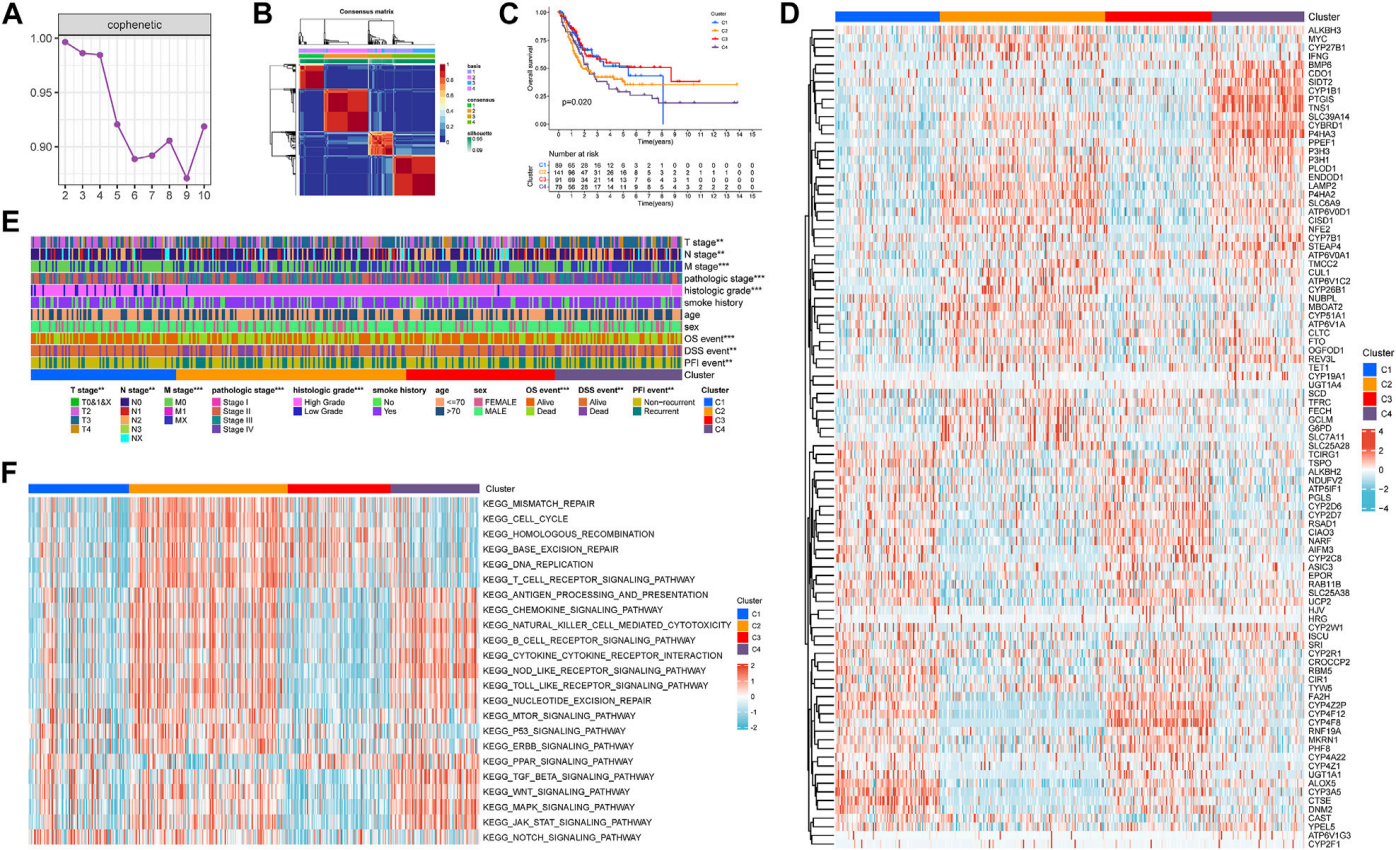
Non-negative matrix factorization clustering of iron metabolism patterns in TCGA-BLCA cohort. **(A)** Cophenetic coefficients of NMF algorithm clustering number from 2–10 **(B)** NMF matrix heatmap when k = 4. **(C)** Kaplan-Meier survival analysis of iron metabolism patterns. **(D)** Expression Heatmap of prognosis-related IMRGs. **(E)** Correlation between iron metabolism pattern and clinicopathological characteristics. **(F)** Heatmap of GSVA analysis among iron metabolism patterns. *p < 0.05; **p < 0.01; ***p < 0.001.

Through the univariate COX regression analysis (*p* < .05) of the TCGA-BLCA patients with integrated survival information and cancer tissue expression profile, 95 IMRGs were selected as prognosis-related genes (Table 1). Then we clustered the TCGA-BLCA cohort by NMF algorithm based on these genes. According to cophenetic coefficients, we decided k = 4 as the best cluster number (Figure 2A). Figure 2B was the NMF matrix heatmap when k = 4, including C1 subtype 89 cases, C2 subtype 141 cases, C3 subtype 91 cases, and C4 subtype 79 cases. Kaplan-Meier survival curves showed that the prognosis of patients in C1 and C3 patterns was better than that of C1 and C2 patterns (*p* = .020) (Figure 2C).

Figure 2D shows the expression of prognosis-related IMRGs in iron metabolic patterns. We also analyzed the clinicopathological differences among distinct iron metabolism patterns (Figure 2E). It was found that the proportion of TNM stages, pathologic stage, histologic grade, OS, DSS, and PFI events was dissimilar among patterns, and the incidence of advanced clinicopathological results in C2 and C4 patterns tended to increase.

Through GSVA analysis, we obtained the rich-concentration pathways among iron metabolism patterns (Figure 2F). We found that C4 pattern was positively related to multiple stromal, carcinogenic, and immune activation related pathways, including TGF-β signaling pathway, WNT signaling pathway, MAPK signaling pathway, JAK-STAT signaling pathway, T cell receptor signaling pathway, chemokine signaling pathway, B cell receptor signaling pathway, cytokine–cytokine receptor interaction, NOD-like receptor signaling pathway, TOLL-like receptor signaling pathway and so on. C2 pattern showed a similar trend to C4 pattern, but C2 was also significantly expressed in a variety of DNA damage repair related pathways. The correlation score of most carcinogenic and immune activation-related pathways was reduced in C1 and C3 patterns. While C3 pattern was also found to exhibit an enriched trend in DNA damage repair related pathways.

### Tumor Immune Microenvironment of Iron Metabolism Patterns

**FIGURE 3:**
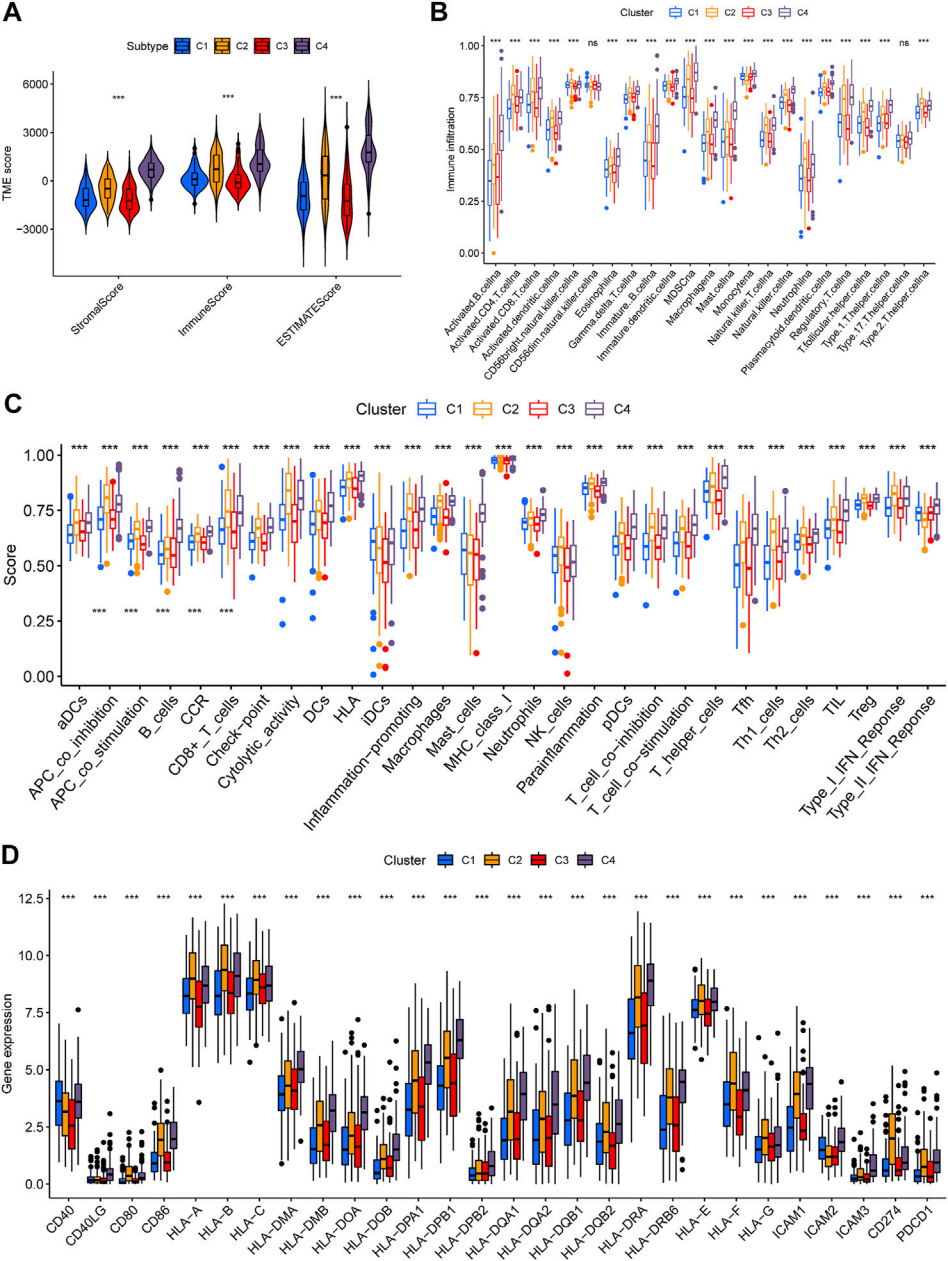
Tumor immune microenvironment of iron metabolism patterns. **(A)** Differences of stromal, immune, and ESTIMATE scores among iron metabolism patterns **(B)** Infiltration of 22 TIICs in iron metabolism patterns. **(C)** Immune-related functions in iron metabolism patterns. **(D)** Difference analysis in MHC molecules, costimulatory molecules, and adhesion molecules of iron metabolism patterns. *p < 0.05; **p < 0.01; ***p < 0.001.

In order to investigate whether there are differences in TIME among distinct iron metabolism patterns, we used ESTIMATE and ssGSEA scores for evaluation. ESTIMATE showed that there were significant differences in the stromal score (*p* < .001), immune score (*p* < .001), and ESTIMATE score (*p* < .001) among the three patterns, of which C4 was the highest, C1 and C3 were the lowest (Figure 3A). Then we analyzed the infiltration differences in immune cells among iron metabolism patterns. The ssGSEA score suggested that the infiltration of all 22 TIICs in iron metabolism patterns was significantly different, among which, the ssGSEA score of TIICs in C1 and C3 patterns was lower and C2 and C4 patterns were higher (Figure 3B). And the enrichment trend of immune-related functions in iron metabolism patterns was similar to that of immune cell infiltration (Figure 3C). Additionally, the expression levels of major histocompatibility complex (MHC) molecules, costimulatory molecules, and adhesion molecules roundly decreased in C1 and C3 patterns (Figure 3D).

### Tumor Somatic Mutation in Iron Metabolism Patterns

**FIGURE 4:**
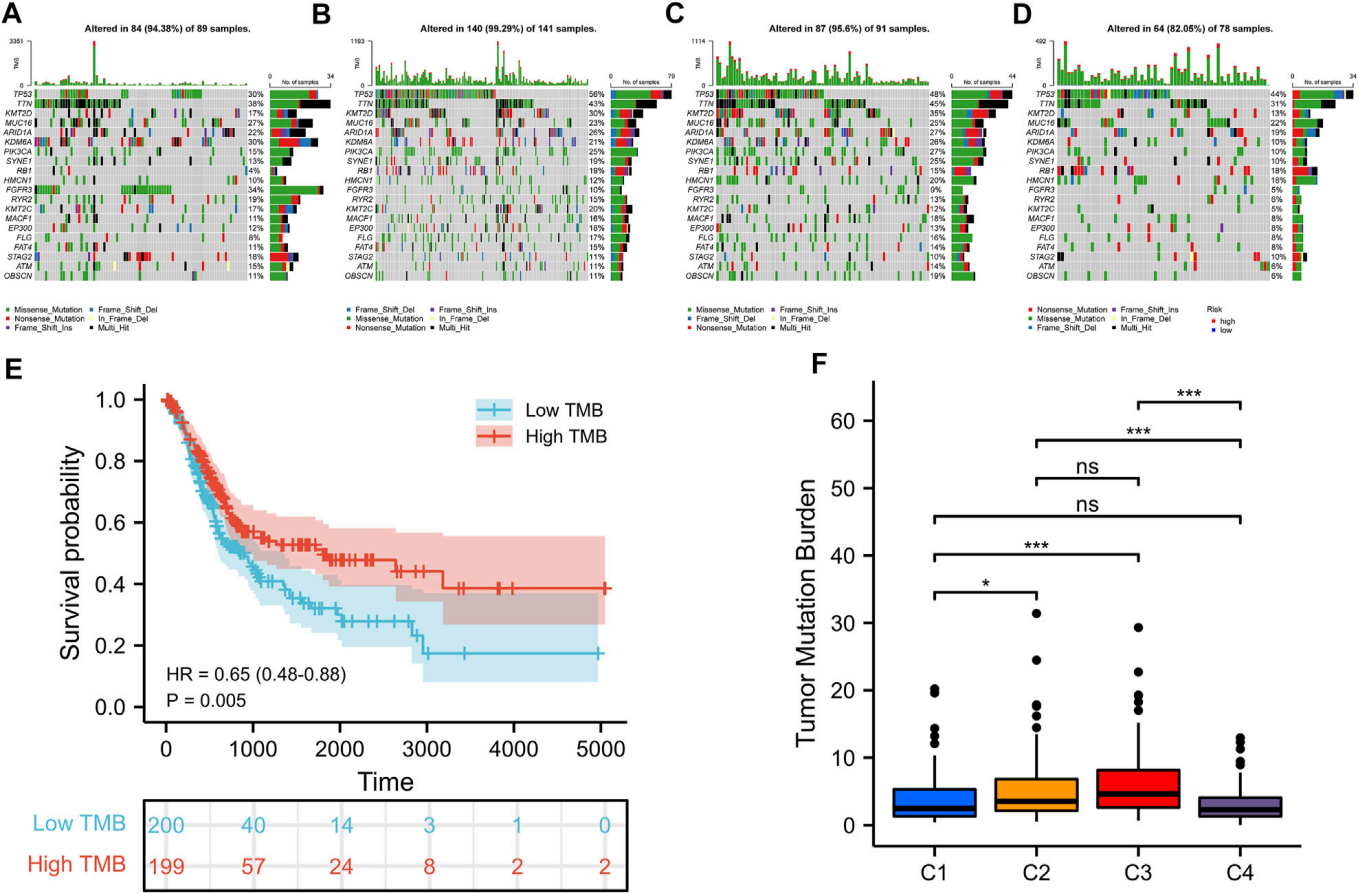
Somatic mutations in distinct iron metabolism patterns. Waterfall plots of 20 genes with the highest mutation rate in C1 pattern **(A)**, C2 pattern **(B)**, C3 pattern **(C)**, and C4 pattern **(D)**. **(E)** Kaplan-Meier survival analysis of TMB in BLCA patients. **(F)** Difference analysis of TMB among different iron metabolism patterns. *p < 0.05; **p < 0.01; ***p < 0.001.

The tumorigenesis frequently occurs after the accumulation of gene mutations (Martincorena and Campbell, 2015). It is also reported that tumor mutation burden (TMB) can be used as a potential prognostic indicator for BLCA (Chan et al., 2019). Consequently, we used the “maftools” R package to show the distribution of somatic mutations and the differences of TMB in various iron metabolism patterns. Through the simple nucleotide variation information of TCGA-BLCA, the mutation spectrum and TMB of each sample was obtained. In BLCA samples, the 20 genes with the highest mutation rate were TP53, TTN, KMT2D, MUC16, ARID1A, KDM6A, PIK3CA, SYNE1, RB1, HMCN1, FGFR3, RYR2, KMT2C, MACF1, EP300, FLG, FAT4, STAG2, ATM and OBSCN (Figures 4A–D). C2 pattern had the highest mutation rate of TP53, while the mutation of TTN and KMT2D mostly happened in C3 pattern. The mutation rates of these three genes in C1 and C4 patterns were significantly reduced. Most gene mutations were missense-mutation. In patients with BLCA, high TMB indicated a better prognosis (hazard ratio [HR] = .65 (.48–.88), *p* = .005) (Figure 4E). Additionally, we found that the TMB of C2 and C3 patterns was significantly upper than that of C1 and C4 patterns (Figure 4F).

### Evaluation of Immunotherapy in Iron Metabolism Patterns

**FIGURE 5:**
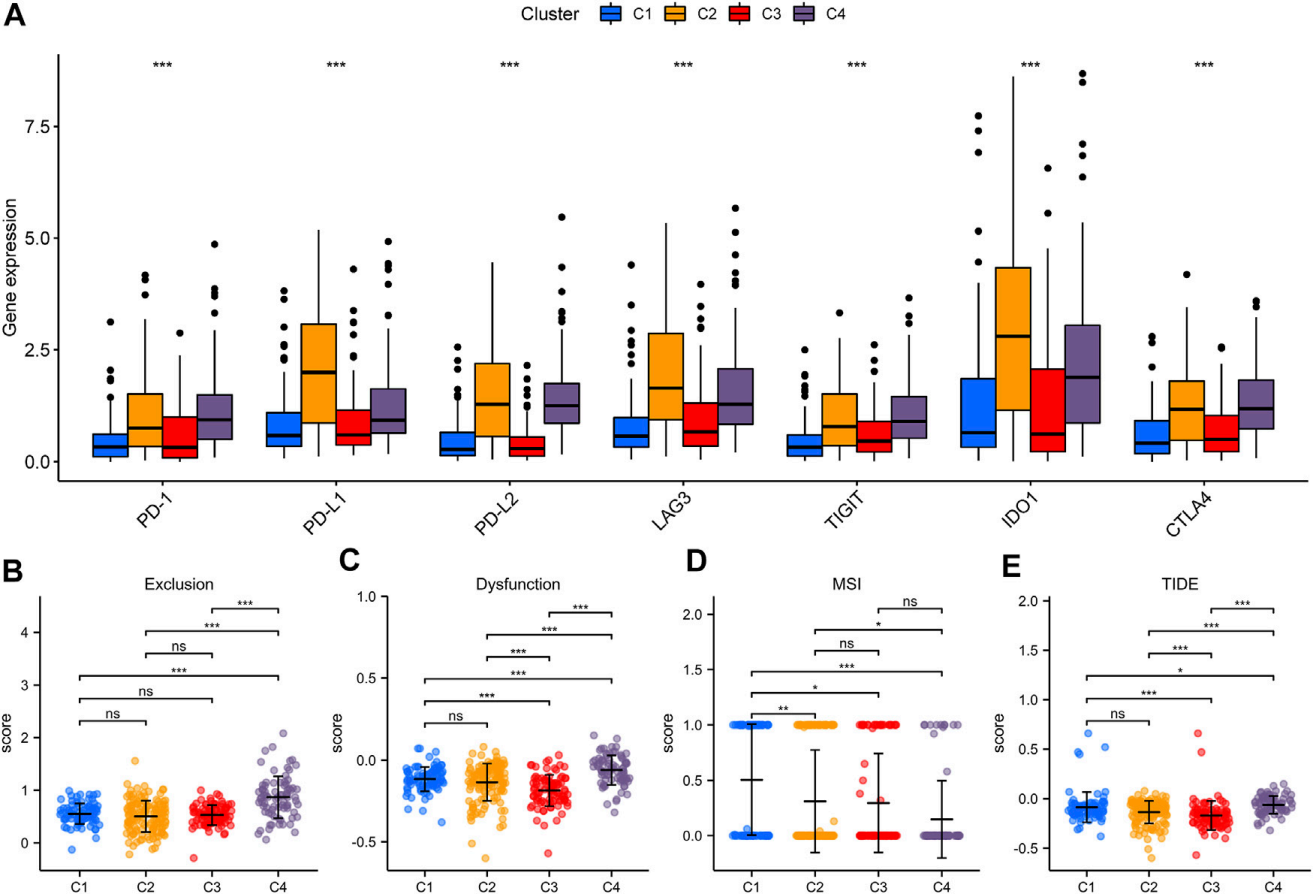
Evaluation of immunotherapeutic therapy in iron metabolism patterns. **(A)** The expression of immune checkpoints in iron metabolism patterns. The comparisons of exclusion score **(B)**, dysfunction score **(C)**, MSI **(D)** and TIDE **(E)** among iron metabolism patterns. *p < 0.05; **p < 0.01; ***p < 0.001.

Lately, immune checkpoint inhibitors (ICIs) have gradually become the second-line treatment for advanced BLCA. Therefore, we analyzed the expression of some immune checkpoints (PDCD1 (PD-1), CD274 (PD-L1), PDCD1LG2 (PD-L2), LAG3, TIGIT, IDO1, and CTLA4) among different iron metabolism patterns to predict the efficacy of immunotherapy (Figure 5A). The expression of all immune checkpoints in C2 and C4 patterns was signally higher than that in the other two patterns. This suggested that C2 and C4 might be more suitable for ICIs treatment. However, the high expression level of immune checkpoints may be related to the formation of the immunosuppressive microenvironment (Dunn et al., 2002). This conclusion was confirmed in Figures 5B,C. C4 immune exclusion score was observably higher than other iron metabolism patterns. The immune dysfunction score of C4 pattern also increased signally, while C3 was the lowest. Moreover, we used the TIDE algorithm to evaluate ICIs response, in which the MSI of C1 and the TIDE of C4 were the highest (Figures 5D,E).

### Chemotherapeutics Drugs Response in Iron Metabolism Patterns

**FIGURE 6:**
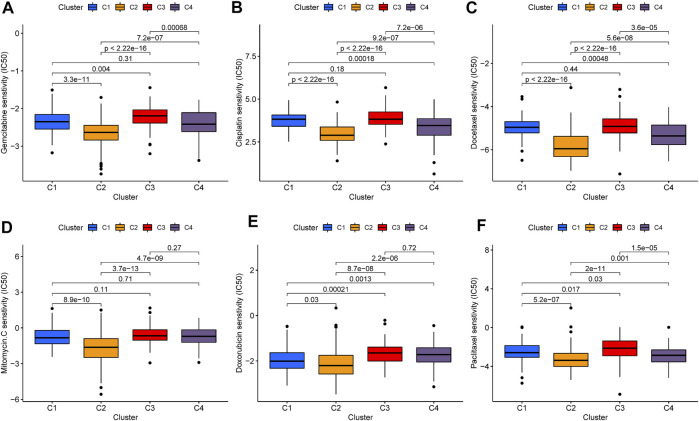
Evaluation of chemotherapy in iron metabolism patterns. The comparisons in IC50 value of Gemcitabine **(A)**, Cisplatin **(B)**, Docetaxel **(C)**, Mitomycin-C **(D)**, Doxorubicin **(E)**, and Paclitaxel **(F)** among iron metabolism patterns.

Chemotherapeutics drugs are widely used in the treatment of BLCA, including intravesical instillation and systemic chemotherapy. Consequently, we evaluated the IC50 values of six commonly used chemotherapeutic drugs (Gemcitabine, Cisplatin, Docetaxel, Mitomycin-C, Doxorubicin, and Paclitaxel) in each sample (Figures 6A–F). Among the six drugs, C2 patterns showed the lowest IC50 value. In Gemcitabine, Cisplatin, Docetaxel, and Paclitaxel, the IC50 value of C4 pattern was also lower than that of C1 and C3 patterns. As for C3 pattern, the IC50 value in Gemcitabine, Doxorubicin, and Paclitaxel was higher than that of C1 pattern. The above results strongly indicated that C2 was the most sensitive to chemotherapeutic drugs, C4 was the second, while C3 was more insensitive to chemotherapeutic drugs.

### Establishment of the IMRG Prognostic Signature in the TCGA-BLCA Cohort

**FIGURE 7:**
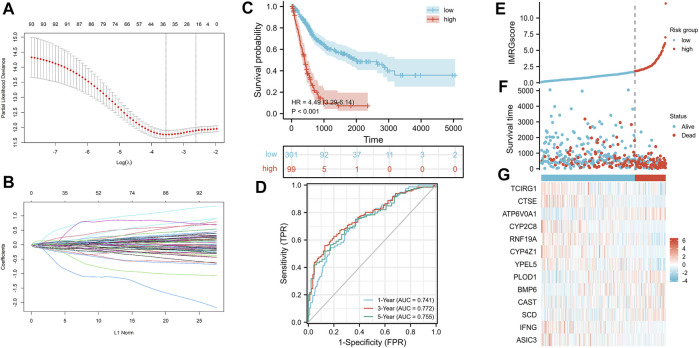
Construction of IMRG signature and prognosis analysis based on the training set. **(A,B)** LASSO regression identified 13 IMRGs. **(C)** Kaplan-Meier survival analysis between IMRGscore-defined groups. **(D)** Time-dependent ROC curves of IMRG signature. **(E)** IMRGscore distribution. **(F)** Survival status map. **(G)** IMRGs expression profiles heatmap.

We selected 400 BLCA patients with explicit, non-zero OS and survival status to establish an IMRG signature from the TCGA database. Then used the LASSO Cox regression model to calculate, and selected an SE higher than the minimum standard to further screen the prognostic genes. Finally, through the multivariate COX regression analysis, we obtained the optimal prognostic signature containing 13 IMRGs, including TCIRG1, CTSE, ATP6V0A1, CYP2C8, RNF19A, CYP4Z1, YPEL5, PLOD1, BMP6, CAST, SCD, IFNG, and ASIC3 (Figures 7A,B). And constructed a formula to evaluate the IMRGscore of each patient: IMRGscore = −(.18775 × TCIRG1 expression) − (.073 × CTSE expression) + (.33856 × ATP6V0A1 expression) − (.37089 × CYP2C8 expression) − (.30306 × RNF19A expression) − (.27636 × CYP4Z1 expression) − (.35016 × YPEL5 expression) + (.17559 × PLOD1 expression) + (.25065 × BMP6 expression) + (.23398 × CAST expression) + (.13313 × SCD expression) − (.52087 × IFNG expression) − (.57726 × ASIC3 expression). And according to the optimal cut-off value (cut point = 1.78265), samples were decomposed into low- and high-risk groups. Kaplan-Meier survival analysis showed that the OS of the low-risk group was longer (hazard ratio [HR] = 4.49 (3.29–6.14), *p* < .001) (Figure 7C). And the AUCs for the 1-, 3-, and 5-year OS survival rates were .741, .772, and .755, respectively (Figure 7D). The risk score distribution, survival status graph, and expression profile heatmap were shown in Figures 7E–G. The proportion of patient deaths was observably positively correlated with the IMRGscore. The expression of ATP6V0A1, PLOD1, BMP6, CAST, and SCD were up-regulated in the high-risk group, while TCIRG1, CTSE, CYP2C8, RNF19A, CYP4Z1, YPEL5, IFNG, and ASIC3 were down-regulated.

### Confirmation of the IMRG Signature in the GSE13507 Cohort

**FIGURE 8:**
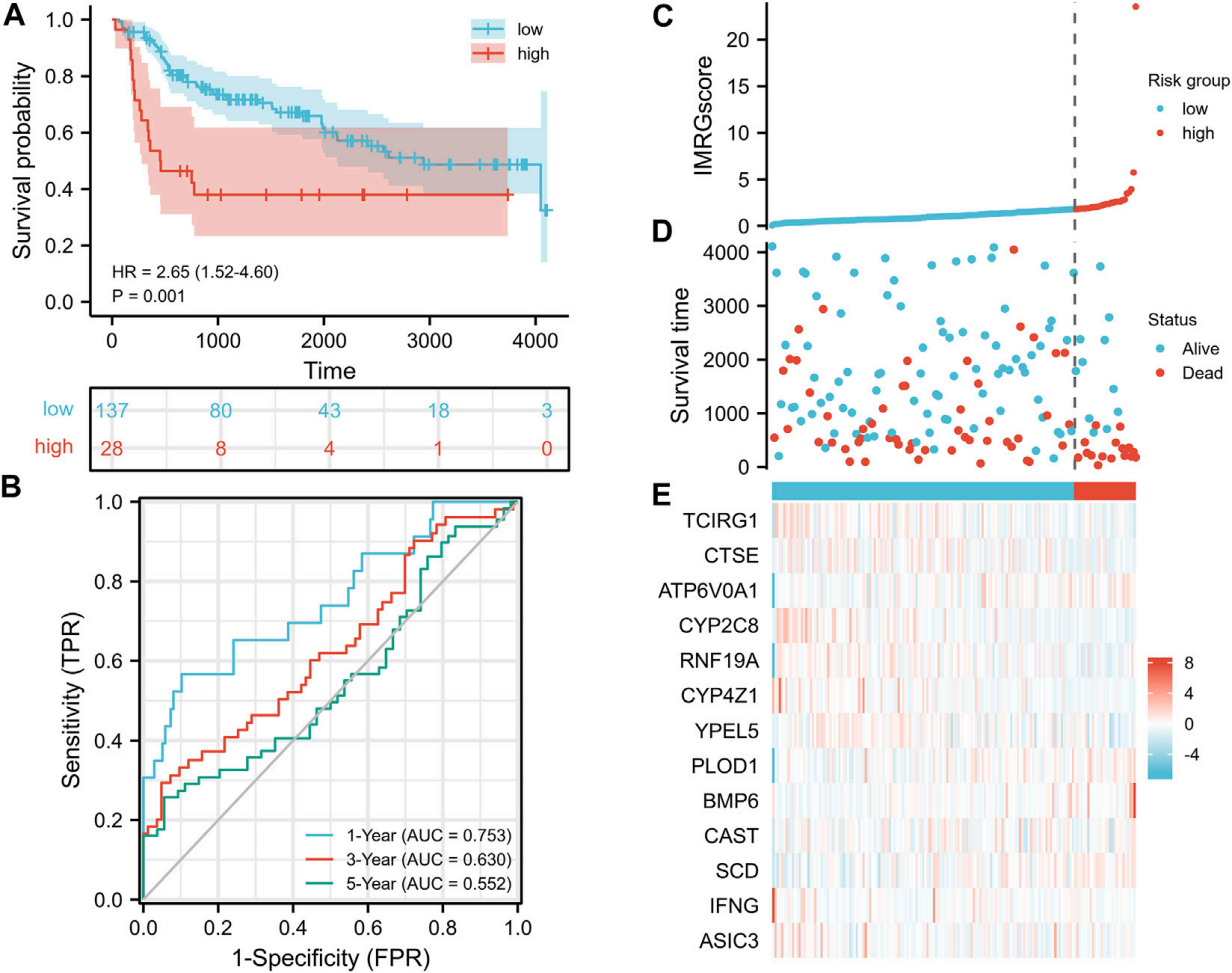
Validation of IMRG signature based on the test set. **(A)** Kaplan-Meier survival analysis between IMRGscore-defined groups. **(B)** Time-dependent ROC curves of IMRG signature. **(C)** IMRGscore distribution. **(D)** Survival status map. **(E)** IMRGs expression profiles heatmap.

As the test set, 165 BLCA samples in the GSE13507 cohort were grouped using the same IMRGscore calculation formula and cut-off value of the train set to validate the applicability and stability of the IMRG signature. Consistent with the above conclusion, patients in the low-risk group had a better OS (hazard ratio [HR] = 2.65 (1.52–4.60), *p* = .001) in the GSE13507 cohort (Figure 8A). The AUCs for the 1-, 3-, and 5-year OS survival rates were .753, .630, and .552, respectively (Figure 8B). The conclusions of the risk score distribution, survival status graph, and expression profile heatmap were consistent with the training set (Figures 8C–E).

### Clinical Relevance of the IMRG Signature

**FIGURE 9:**
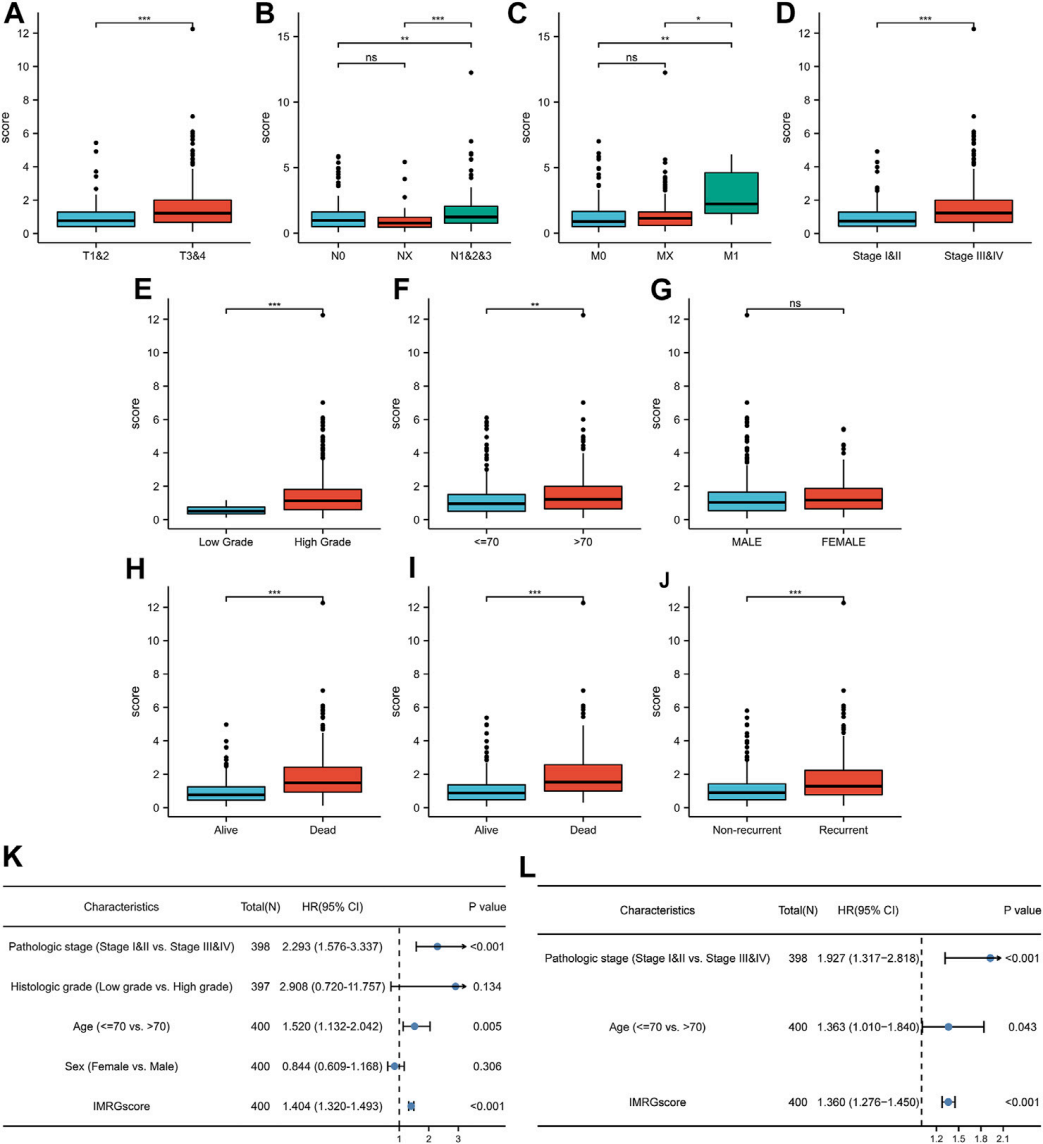
Clinical relevance of IMRG signature. Correlation between IMRGscore and clinicopathological characteristics, including T stage **(A)**, N stage **(B)**, M stage **(C)**, pathologic stage **(D)**, histologic grade **(E)**, age **(F)**, gender **(G)**, OS event **(H)**, DSS event **(I)**, and PFI event **(J)**. Univariate **(K)** and multivariate **(L)** Cox regression analysis of risk-group and clinicopathological characteristics. *p < 0.05; **p < 0.01; ***p < 0.001.

To further supplement the clinical application value of the IMRG prognostic signature, we integrated the significant differences in IMRGscore among distinct subgroups of BLCA patients with clinicopathological characteristics (Figure 9A). Heatmap indicated that the advanced TNM stages, pathologic stage, histologic grade, aging, and worse OS, DSS, and PFI events had an elevated trend in IMRGscore.

Since the significant correlation between signature and clinicopathological stage, we determined whether the IMRGscore was a clinically independent predictor of BLCA patients (Figures 9B,C). Univariate Cox regression analysis showed that advanced pathologic stage (*p* < .001), aging (*p* = .005), and higher IMRGscore (*p* < .001) were unfavorable factors for OS. After performing the multivariate Cox regression analysis, we confirmed that the IMRGscore was an independent prognostic parameter.

### Establishment of a Nomogram Based on the IMRG Signature

**FIGURE 10:**
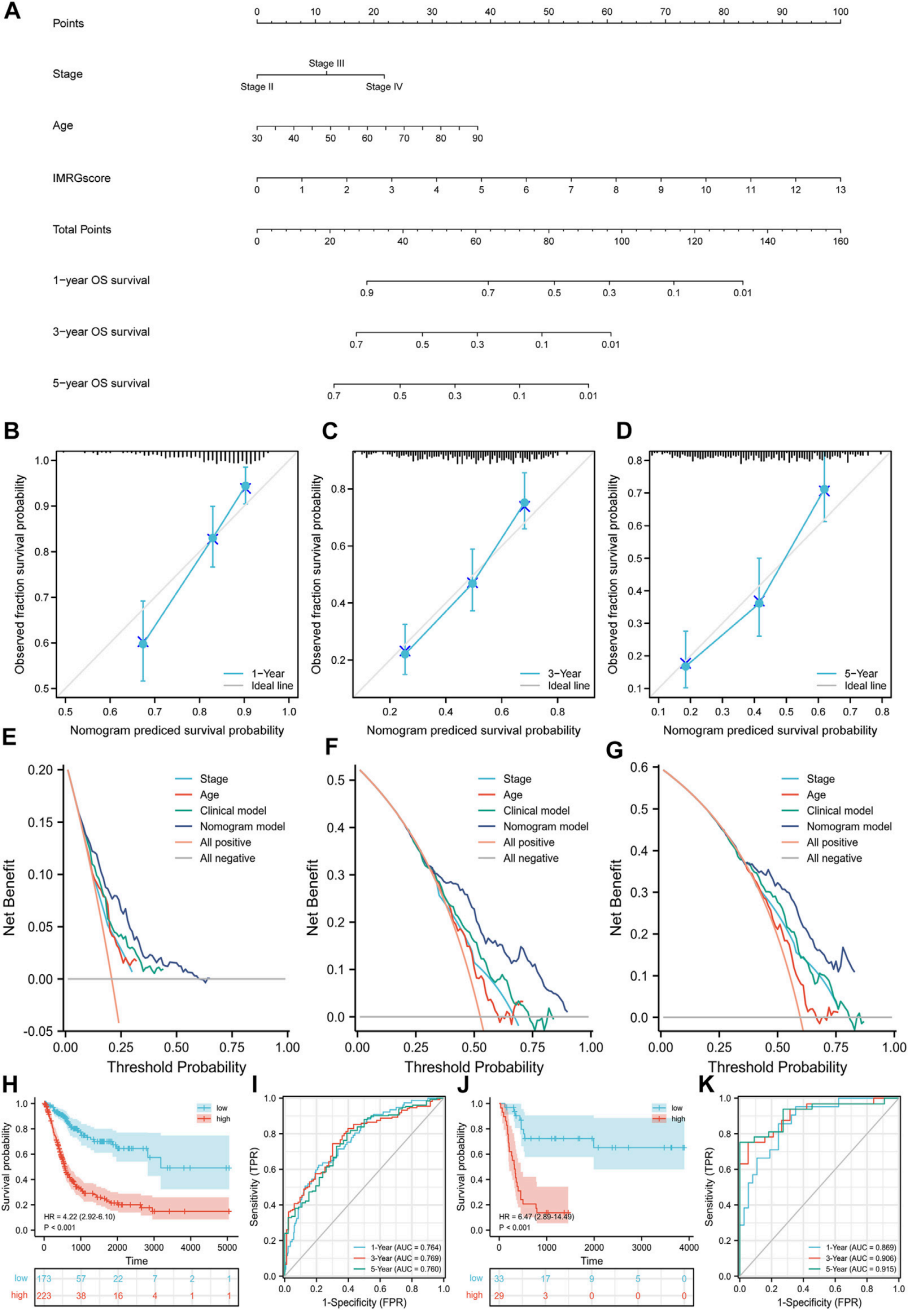
Nomogram model based on clinicopathological characteristics and IMRGscore. **(A)** Nomogram for predicting the probability of OS over 1, 3, and 5 years. **(B–D)** Calibration curves for predicting the fitness of the nomogram model in 1, 3, and 5 years. **(E–G)** DCA curves based on three predictors of 1, 3, and 5 years. **(H)** Kaplan-Meier analysis of nomogram model in the TCGA-BLCA cohort. **(I)** Time-dependent ROC curves of nomogram model in the TCGA-BLCA cohort. **(J)** Kaplan-Meier analysis of nomogram model in the GSE13507 cohort. **(K)** Time-dependent ROC curves of nomogram model in the GSE13507 cohort.

According to the above result from univariate Cox regression analysis of the TCGA-BLCA cohort, we established a nomogram model containing pathologic stage, age, and IMRGscore (Figure 10A). After removing the patients without complete information and the subgroups of variables with few samples, a total of 362 patients were included. We standardized each variable with a score from 0 to 100 and summed the scores to obtain the total number of points for each BLCA patient. The predicted 1-, 3-, and 5-year survival probabilities of each patient were standardized according to the relationship between the positions along with the prognosis and total points axes. The C-index reached 0.694 (95% CI: 0.653–0.735). Figures 10B–D suggested that the nomogram model predicted that the prognosis results of TCGA-BLCA patients would fit well with the actual prognosis results. Besides, DCA curves revealed that the signature provided patients with a stable and significant net benefit in BLCA patients (Figures 10E–G). Then based on the nomogram model, we built the Kaplan-Meier survival curve and the time-dependent ROC curves. In the TCGA-BLCA cohort, we divided samples into high- and low-risk groups with the optimal cut-off value (cut point = −.122532). Patients in the high-risk group showed a significantly poor OS (hazard ratio [HR] = 4.22 (2.92–6.10), *p* < .001, Figure 10H). The AUCs for the 1-, 3-, and 5-year OS survival rates were .764, .769, and .760, respectively (Figure 10I). Additionally, we verified the nomogram model in the GSE13507 cohort. The cut-off value was consistent with the train set. Kaplan-Meier survival analysis showed that patients in the low-risk group had a better OS than those in the high-risk group (hazard ratio [HR] = 6.47 (2.89–14.49), *p* < .001, Figure 10J), and the AUCs were .896, .906, and .915 (Figure 10K).

### GSEA

**FIGURE 11:**
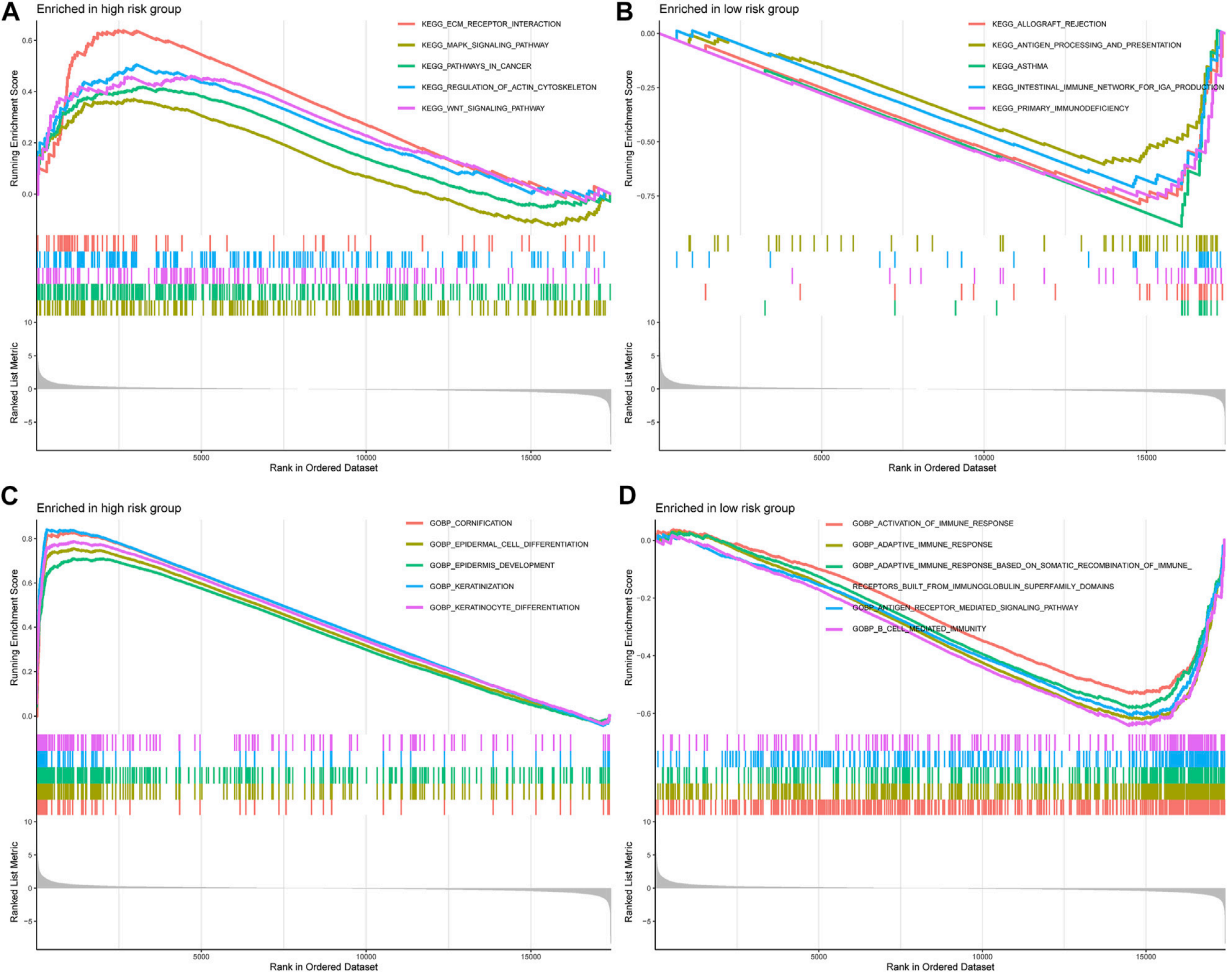
Correlation between IMRG signature and biological functions. KEGG results of the high-risk group **(A)** and low-risk group **(B)**. GOBP results of the high-risk group **(C)** and low-risk group **(D)**.

To further comprehend the effect of IMRGs expression on the biological characteristics of BLCA, we carried on GSEA analysis in IMRGscore-defined groups (Figures 11A,B). The Kyoto Encyclopedia of Genes and Genomes (KEGG) results revealed that in the high-risk group, the main enrichment pathways were ECM receptor interaction, regulation of actin cytoskeleton, MAPK signaling pathway, WNT signaling pathway, pathways in cancer. While the low-risk group is mainly concentrated in allograft rejection, asthma, primary immunodeficiency, and so on. Furthermore, Figures 11C,D showed the enrichment of the high- and low-risk groups in the Gene Ontology biological process (GOBP). We found that the low-risk group was enriched in multiple immune functions, such as activation of immune response, adaptive immune response, B cell-mediated immunity, and so on.

### Correlation Between Tumor Immune Microenvironment and IMRG Signature

**FIGURE 12:**
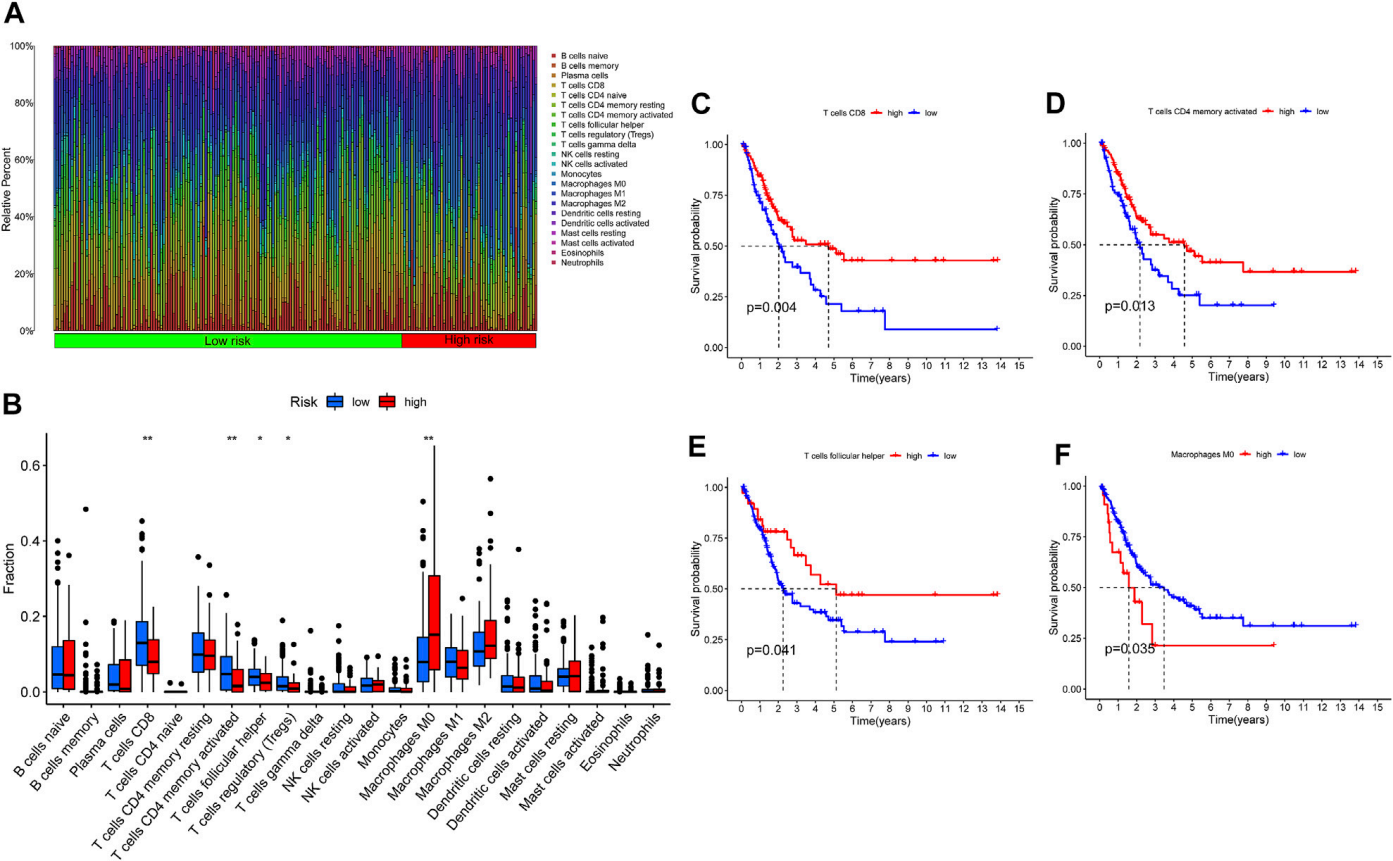
Tumor immune microenvironment of IMRG signature. **(A)** The proportion of 22 TIICs in BLCA. **(B)** Infiltration of 22 TIICs in low- and high-risk groups. Correlation between the infiltration level of TIICs (CD8 T cells **(C)**, activated CD4 memory T cells **(D)**, follicular helper T cells **(E)**, and M0 macrophages **(F)** and prognosis of patients with BLCA. *p < 0.05; **p < 0.01.

As we mentioned before, iron metabolism is closely related to TIME. Based on the CIBERSORT algorithm, we calculated the proportion of 22 TIICs in each TCGA-BLCA sample (Figure 12A). After selecting samples with significant immune cell fraction results (*p* < .05), 195 samples were included in the difference analysis, including 141 cases in the low-risk group and 54 cases in the high-risk group. Then the difference in the proportion of TIICs between the IMRGscore-defined groups was shown in Figure 12B. It was found that the fraction of CD8 T cells, activated CD4 memory T cells, follicular helper T cells (TFH) and regulatory T cells (Treg) in the low-risk group was significantly higher. In contrast, the fraction of M0 macrophages was upper in the high-risk group. Furthermore, high levels of CD8 T cells (*p* = .004), activated CD4 memory T cells (*p* = .013) and TFH (*p* = .041) were observably associated with better OS (Figures 12C–E), while increased M0 macrophages (*p* = .035) indicated a poor OS (Figure 12F).

## Discussion

Bladder cancer is a heterogeneous malignancy. Patients with BLCA generally show different prognoses because of the molecular discrepancy (Knowles and Hurst, 2015; Guo and Czerniak, 2019). At present, it is generally believed that clinical or pathological stages were insufficient to predict the prognosis of patients with BLCA (Konety, 2006; Rosenberg et al., 2013). Therefore, it is essential to develop a more accurate and efficient model to predict the survival prognosis for patients. In recent years, some studies have found iron involvement in the appearance and progression of cancers. Abundant researches have revealed that iron metabolism is involved in the entire process of cancer progression. Murata M. demonstrated that iron released from the damaged transferrin could mediate the Fenton reaction and produce ROS, which contributes to the carcinogenic process in multiple ways (Murata, 2018). A cross-sectional study found that the serum iron concentration in patients with BLCA was lower than that in the control group (Mazdak et al., 2010). However, studies on the potential function of iron metabolism in the treatment and prognosis of BLCA are scarce.

In this study, patients in the TCGA-BLCA cohort were divided into four iron metabolism patterns based on the expression of prognosis-related IMRGs. Survival analysis suggested that C1 and C3 had a better prognosis. Our results also showed that C2 and C4 patterns have higher enrichment scores in multiple carcinogenic and immune activation-related pathways. For instance, abnormal activation of NOD-like receptors (NLRs) occurs in various cancers, coordinates the tumor immune microenvironment, and promotes angiogenesis, cancer cell stem cells, and chemotherapy resistance, thereby enhancing tumor risk (Liu et al., 2019). Toll-like receptor (TLR) is a transmembrane pattern recognition receptor that detects and defends microbial pathogens through the innate immune response (Brennan and Gilmore, 2018). The activation of the Notch signaling pathway can be seen in most components of the tumor microenvironment (TME), such as angiogenesis, tumor stem cell maintenance, immune infiltration, or therapeutic resistance (Meurette and Mehlen, 2018). Besides, C2 and C4 iron metabolism patterns were highly infiltrated by immune cells, and the expression of MHC genes was highly elevated. These characteristics are consistent with an immune-inflamed phenotype. On the other hand, C1 and C3 are suspected to be immune-desert phenotypes due to lack of immune infiltration and antigen presentation. Additionally, we found that multiple immune checkpoints (PD-1, PD-L1, PD-L2, LAG3, TIGIT, IDO1, and CTLA4) were highly expressed in C2 and C4 patterns, which might indicate that immunotherapy has a better effect on them. Furthermore, studies have shown that the high-level expression of immune checkpoint genes might be a stimulative to the immunosuppressive microenvironment and led to the immune escape of tumor cells (Dunn et al., 2002). The TIDE algorithm confirmed this conclusion. Therefore, we speculate that the reason why C2 and C4 patterns do not show a better prognosis of immunoinflammatory phenotype is that the antitumor effect based on the activated immune pathway and high infiltration level of T cells were eliminated by the formation of the immunosuppressive microenvironment. The above evidence proved that iron metabolism is of great significance in shaping various TME landscapes in BLCA.

Somatic mutation is not only the driving factor of cancer, but also the guiding basis for diagnosis and treatment. The three genes with the highest mutation rate in BLCA were TP53, TTN, and KMT2D. The mutation rate of TP53 in C2 iron metabolism pattern was the highest, while in C1 pattern was the lowest. In C2 and C3 patterns, the incidence of TTN and KMT2D mutations was significantly higher. Detection of TP53 mutation was conducive to estimating the high risk of early lesions (Olivier et al., 2010). Single TTN gene mutation often indicated high TMB (Oh et al., 2020). The mutation of epigenetic regulator KMT2D was a biomarker of poor prognosis in some cancers (Ferrero et al., 2020). Additionally, we found that high TMB suggested that patients with BLCA had a better prognosis through the TCGA database. Consistently, the TMB of C1 and C4 patterns was observably decreased. These results show that iron metabolism has a complex interaction with somatic mutation.

Chemotherapy is still one of the main treatments for BLCA. According to the latest guidelines of the European Association of Urology (EAU), all muscle-invasive bladder cancer (MIBC) patients with physical conditions can apply platinum-based neoadjuvant chemotherapy before operation (Witjes et al., 2021). This study investigated the efficacy of six common chemotherapeutic drugs on iron metabolism mode, including Gemcitabine, Cisplatin, Docetaxel, Mitomycin-C, Doxorubicin, and Paclitaxel. The results showed that C2 pattern was the most sensitive to these chemotherapeutic drugs, while C3 pattern was the most insensitive. This provided a reference basis for the selection of clinical chemotherapy drugs.

Due to the effect of iron metabolism on the tumorigenesis and progression of cancer, it is of great importance to establish an IMRG signature for predicting the prognosis of patients with BLCA. Herein, we applied stepwise regression analysis to compose a clinical prognostic signature for BLCA patients with 13 IMRGs (TCIRG1, CTSE, ATP6V0A1, CYP2C8, RNF19A, CYP4Z1, YPEL5, PLOD1, BMP6, CAST, SCD, IFNG, and ASIC3). A test set was utilized to confirm its accuracy and stability. Moreover, the IMRGscore was elevated in multiple advanced clinicopathological stages. And multivariate Cox regression analysis verified that IMRGscore was an independent prognostic index of BLCA patients. Finally, we combined some clinicopathological features, including pathologic stage, age, and IMRGscore to construct a nomogram that accurately predicted the prognosis of patients with BLCA. The accuracy and clinical contributions were verified by calibration analysis and DCA.

Several studies have shown that these 13 IMRGs are closely related to cancers, and some of these genes have been confirmed about BLCA. TCIRG1, one of the V-ATPase subunits, is abnormally overexpressed in patients with recurrent hepatocellular carcinoma, and enhances the ability of metastasis by regulating the growth, death, and epithelial to mesenchymal transition of cancer cells (Yang et al., 2018). A retrospective study suggests that CTSE can be used as an independent prognostic marker for NMIBC, so as to guide the treatment of patients (Lin et al., 2001). ATP6V0A1was demonstrated that it could enhance the fusion of autophagosomes and lysosomes, up-regulate autophagy volume accumulation, and finally induce autophagic cell death (Hsin et al., 2012). CYP2C8 can be regulated by GAS5/miR-382-3p in hepatocellular carcinoma and play an anticancer role (Li and Chen, 2020). RNF19A was confirmed to be overexpressed in non-small cell lung cancer, which plays a carcinogenic role by destroying the function of p53 (Cheng et al., 2021). CYP4Z1 was confirmed to be highly expressed in BLCA and positively correlated with the progression of histologic grade and pathologic stage (Al-Saraireh et al., 2021). YPEL5 was found to be inhibited by METTL3-m6A (N6-methyladenosine)-YTHDF2 axis in colorectal cancer, promoting the growth and metastasis of tumor (Zhou et al., 2021). The expression of PLOD1 was an independent prognostic factor in BLCA patients, and downregulated by inhibitor could significantly reduce the invasiveness of BLCA cells (Yamada et al., 2019). The expression of BMP6, a key endogenous regulator of iron metabolism, was affected by Med19, which could promote bone metastasis and invasiveness of bladder cancer (Andriopoulos Jr et al., 2009; Wen et al., 2013). Public clinical data also confirm that BMP6 is a prognostic marker for bladder cancer (Yuen et al., 2012). Calpastatin (CAST) is involved in many important physiological processes, including cell cycle, ECM, cancer cell proliferation, metastasis, and apoptosis (Nian and Ma, 2021). SCD can protect cancer cells from oxidative stress and ferroptosis through mediated lipogenesis in prostate cancer with over-activation of PI3K-AKT-mTOR signaling (Yi et al., 2020). SCD has also been shown to reduce proliferation and invasion of BLCA cells when inhibited (Piao et al., 2019). Iron metabolism can affect innate immune response by affecting IFNG mediated immune response pathway in macrophages (Nairz et al., 2014). And IFNG was demonstrated to inhibit the activity of bladder cancer stem cells (Qiu et al., 2020). ASIC3 has an H+ gating function, which promotes the acid-induced epithelial-mesenchymal transition in pancreatic cancer cells (Zhu et al., 2017).

GSEA analysis showed that several cancer-related pathways were enriched in the high-risk group. The unique biochemical and biophysical properties of ECM when it is dysregulated are the key drivers of cancer progression (Walker et al., 2018). The MAPK signaling pathway is considered to be related to cell proliferation, differentiation, migration, aging, and apoptosis (Sun et al., 2015). The Wnt signaling pathway is an important driving factor to maintain tissue development and homeostasis. Abnormal Wnt signaling will cause the occurrence and progression of many cancers by affecting cancer stem cells (Duchartre et al., 2016). Furthermore, CIBERSORT analysis indicated that several TIICs with differential infiltration levels had significant correlations between the prognosis of BLCA patients, and played a regulatory role in the balance of iron metabolism. After being activated by immunotherapy, CD8 T cells can enhance ferroptosis-specific lipid peroxidation and increase ferroptosis in tumor cells, which contributes to the anti-tumor effect (Wang et al., 2019). Macrophages are the regulatory hub of iron metabolism. Macrophages phagocytize and degrade aging and damaged erythrocytes to recover iron, and also have the ability to release iron. The accumulation of M2 macrophages is often associated with poor prognosis, and M2 macrophages possess iron release characteristics (Recalcati et al., 2010). Therefore, the accumulation of M2 macrophages may further aggravate the disorder of iron metabolism.

Our research has obvious advantages in the study of iron metabolism characteristics and the clinical application of BLCA. First, we divided BLCA patients into distinct iron metabolism landscapes to further confirm the relationship between iron metabolism and TME and somatic mutations. At the same time, we also verified that iron metabolism landscapes had guiding significance for chemotherapy drugs and immunotherapy selection. Next, we established the IMRG prognostic signature and proved IMRGscore was an independent prognostic factor for BLCA patients, and it also had the predictive ability for clinicopathological characteristics. At present, our research is still insufficient. First, it is a retrospective study. Deviations in variables such as clinicopathological characteristics of patients most likely exist. Second, our prognostic signature depends on gene expression and does not take into account the effects of gene mutation, methylation, or other factors on the prognosis of BLCA patients. Finally, the prognostic signature can be incorporated into large sample prospective studies to further verify its clinical value.

## Data Availability

Raw data for this study were generated at the TCGA database with the cancer type of BLCA. The datasets used and/or analyzed during the current study are available from the GEO database (GSE13507). Derived data supporting the findings are available from the corresponding author (WS) on reasonable request.

## References

[B1] Al-Saraireh Y. M. Alshammari F. O. F. O. Youssef A. M. M. Al-Sarayreh S. Almuhaisen G. H. Alnawaiseh N. (2021). Profiling of CYP4Z1 and CYP1B1 Expression in Bladder Cancers. Sci. Rep. 11 (1), 5581. 10.1038/s41598-021-85188-4 33692504 PMC7946900

[B2] Andrews N. C. (2008). Forging a Field: the golden Age of Iron Biology. Blood 112 (2), 219–230. 10.1182/blood-2007-12-077388 18606887 PMC2442739

[B3] Andriopoulos Jr B. Jr. Corradini E. Xia Y. Faasse S. A. Chen S. Grgurevic L. (2009). BMP6 Is a Key Endogenous Regulator of Hepcidin Expression and Iron Metabolism. Nat. Genet. 41 (4), 482–487. 10.1038/ng.335 19252486 PMC2810136

[B4] Babjuk M. Burger M. Compérat E. M. Gontero P. Mostafid A. H. Palou J. (2019). European Association of Urology Guidelines on Non-muscle-invasive Bladder Cancer (TaT1 and Carcinoma *In Situ*) - 2019 Update. Eur. Urol. 76 (5), 639–657. 10.1016/j.eururo.2019.08.016 31443960

[B5] Battaglia A. M. Chirillo R. Aversa I. Sacco A. Costanzo F. Biamonte F. (2020). Ferroptosis and Cancer: Mitochondria Meet the "Iron Maiden" Cell Death. Cells 9 (6), 1505. 10.3390/cells9061505 PMC7349567 32575749

[B6] Berdik C. (2017). Unlocking Bladder Cancer. Nature 551 (7679), S34–s35. 10.1038/551S34a 29117159

[B7] Bialasek M. Kubiak M. Gorczak M. Braniewska A. Kucharzewska-Siembieda P. Krol M. (2019). Exploiting Iron-Binding Proteins for Drug Delivery. J. Physiol. Pharmacol. 70 (5). 10.26402/jpp.2019.5.03 31889039

[B8] Brennan J. J. Gilmore T. D. (2018). Evolutionary Origins of Toll-like Receptor Signaling. Mol. Biol. Evol. 35 (7), 1576–1587. 10.1093/molbev/msy050 29590394

[B9] Brunet J.-P. Tamayo P. Golub T. R. Mesirov J. P. (2004). Metagenes and Molecular Pattern Discovery Using Matrix Factorization. Proc. Natl. Acad. Sci. 101 (12), 4164–4169. 10.1073/pnas.0308531101 15016911 PMC384712

[B10] Chan T. A. Yarchoan M. Jaffee E. Swanton C. Quezada S. A. Stenzinger A. (2019). Development of Tumor Mutation burden as an Immunotherapy Biomarker: Utility for the Oncology Clinic. Ann. Oncol. 30 (1), 44–56. 10.1093/annonc/mdy495 30395155 PMC6336005

[B11] Cheng Y. Hu Y. Wang H. Zhao Z. Jiang X. Zhang Y. (2021). Ring finger Protein 19A Is Overexpressed in Non-small Cell Lung Cancer and Mediates P53 Ubiquitin-Degradation to Promote Cancer Growth. J. Cel Mol Med 25 (16), 7796–7808. 10.1111/jcmm.16674 PMC8358885 34184814

[B12] Duchartre Y. Kim Y.-M. Kahn M. (2016). The Wnt Signaling Pathway in Cancer. Crit. Rev. Oncology/Hematology 99, 141–149. 10.1016/j.critrevonc.2015.12.005 PMC5853106 26775730

[B13] Dufès C. Al Robaian M. Somani S. (2013). Transferrin and the Transferrin Receptor for the Targeted Delivery of Therapeutic Agents to the Brain and Cancer Cells. Ther. Deliv. 4 (5), 629–640. 10.4155/tde.13.21 23647279

[B14] Dunn G. P. Bruce A. T. Ikeda H. Old L. J. Schreiber R. D. (2002). Cancer Immunoediting: from Immunosurveillance to Tumor Escape. Nat. Immunol. 3 (11), 991–998. 10.1038/ni1102-991 12407406

[B15] Ferrero S. Rossi D. Rinaldi A. Bruscaggin A. Spina V. Eskelund C. W. (2020). KMT2D Mutations and TP53 Disruptions Are Poor Prognostic Biomarkers in Mantle Cell Lymphoma Receiving High-Dose Therapy: a FIL Study. Haematologica 105 (6), 1604–1612. 10.3324/haematol.2018.214056 31537689 PMC7271566

[B16] Fonseca-Nunes A. Jakszyn P. Agudo A. (2014). Iron and Cancer Risk-A Systematic Review and Meta-Analysis of the Epidemiological Evidence. Cancer Epidemiol. Biomarkers Prev. 23 (1), 12–31. 10.1158/1055-9965.Epi-13-0733 24243555

[B17] Geeleher P. Cox N. Huang R. S. (2014). pRRophetic: an R Package for Prediction of Clinical Chemotherapeutic Response from Tumor Gene Expression Levels. PLoS One 9 (9), e107468. 10.1371/journal.pone.0107468 25229481 PMC4167990

[B18] Guo C. C. Czerniak B. (2019). Bladder Cancer in the Genomic Era. Arch. Pathol. Lab. Med. 143 (6), 695–704. 10.5858/arpa.2018-0329-RA 30672335

[B19] Hassannia B. Vandenabeele P. Vanden Berghe T. (2019). Targeting Ferroptosis to Iron Out Cancer. Cancer Cell 35 (6), 830–849. 10.1016/j.ccell.2019.04.002 31105042

[B20] Hsin I.-L. Sheu G.-T. Jan M.-S. Sun H.-L. Wu T.-C. Chiu L.-Y. (2012). Inhibition of Lysosome Degradation on Autophagosome Formation and Responses to GMI, an Immunomodulatory Protein fromGanoderma Microsporum. Br. J. Pharmacol. 167 (6), 1287–1300. 10.1111/j.1476-5381.2012.02073.x 22708544 PMC3504994

[B21] Jiang P. Gu S. Pan D. Fu J. Sahu A. Hu X. (2018). Signatures of T Cell Dysfunction and Exclusion Predict Cancer Immunotherapy Response. Nat. Med. 24 (10), 1550–1558. 10.1038/s41591-018-0136-1 30127393 PMC6487502

[B22] Jung M. Mertens C. Tomat E. Brüne B. (2019). Iron as a Central Player and Promising Target in Cancer Progression. Int. J. Mol. Sci. 20 (2), 273. 10.3390/ijms20020273 PMC6359419 30641920

[B23] Knowles M. A. Hurst C. D. (2015). Molecular Biology of Bladder Cancer: New Insights into Pathogenesis and Clinical Diversity. Nat. Rev. Cancer 15 (1), 25–41. 10.1038/nrc3817 25533674

[B24] Konety B. R. (2006). Molecular Markers in Bladder Cancer: a Critical Appraisal. Urol. Oncol. Semin. Original Invest. 24 (4), 326–337. 10.1016/j.urolonc.2005.11.023 16818187

[B25] Li K. Chen Y. (2020). CYP2C8 Regulated by GAS5/miR-382-3p Exerts Anti-cancerous Properties in Liver Cancer. Cancer Biol. Ther. 21 (12), 1145–1153. 10.1080/15384047.2020.1840886 33180658 PMC7722789

[B26] Lin C. K. Lai K. H. Lo G. H. Cheng J. S. Hsu P. I. Mok K. T. (2001). Cathepsin E and Subtypes of Intestinal Metaplasia in Carcinogenesis of the Human Stomach. Zhonghua Yi Xue Za Zhi (Taipei) 64 (6), 331–336. 11534800

[B27] Liu P. Lu Z. Liu L. Li R. Liang Z. Shen M. (2019). NOD-like Receptor Signaling in Inflammation-Associated Cancers: From Functions to Targeted Therapies. Phytomedicine 64, 152925. 10.1016/j.phymed.2019.152925 31465982

[B28] Manz D. H. Blanchette N. L. Paul B. T. Torti F. M. Torti S. V. (2016). Iron and Cancer: Recent Insights. Ann. N.Y. Acad. Sci. 1368 (1), 149–161. 10.1111/nyas.13008 26890363 PMC4870095

[B29] Martincorena I. Campbell P. J. (2015). Somatic Mutation in Cancer and normal Cells. Science 349 (6255), 1483–1489. 10.1126/science.aab4082 26404825

[B30] Mazdak H. Yazdekhasti F. Movahedian A. Mirkheshti N. Shafieian M. (2010). The Comparative Study of Serum Iron, Copper, and Zinc Levels between Bladder Cancer Patients and a Control Group. Int. Urol. Nephrol. 42 (1), 89–93. 10.1007/s11255-009-9583-4 19548109

[B31] Meurette O. Mehlen P. (2018). Notch Signaling in the Tumor Microenvironment. Cancer Cell 34 (4), 536–548. 10.1016/j.ccell.2018.07.009 30146333

[B32] Mou Y. Wang J. Wu J. He D. Zhang C. Duan C. (2019). Ferroptosis, a New Form of Cell Death: Opportunities and Challenges in Cancer. J. Hematol. Oncol. 12 (1), 34. 10.1186/s13045-019-0720-y 30925886 PMC6441206

[B33] Murata M. (2018). Inflammation and Cancer. Environ. Health Prev. Med. 23 (1), 50. 10.1186/s12199-018-0740-1 30340457 PMC6195709

[B34] Nairz M. Haschka D. Demetz E. Weiss G. (2014). Iron at the Interface of Immunity and Infection. Front. Pharmacol. 5, 152. 10.3389/fphar.2014.00152 25076907 PMC4100575

[B35] Nian H. Ma B. (2021). Calpain-calpastatin System and Cancer Progression. Biol. Rev. 96 (3), 961–975. 10.1111/brv.12686 33470511

[B36] Oh J.-H. Jang S. J. Kim J. Sohn I. Lee J.-Y. Cho E. J. (2020). Spontaneous Mutations in the Single TTN Gene Represent High Tumor Mutation burden. Npj Genom. Med. 5, 33. 10.1038/s41525-019-0107-6 PMC7424531 32821429

[B37] Olivier M. Hollstein M. Hainaut P. (2010). TP53 Mutations in Human Cancers: Origins, Consequences, and Clinical Use. Cold Spring Harbor Perspect. Biol. 2 (1), a001008. 10.1101/cshperspect.a001008 PMC2827900 20182602

[B38] Piao C. Cui X. Zhan B. Li J. Li Z. Li Z. (2019). Inhibition of Stearoyl CoA Desaturase-1 Activity Suppresses Tumour Progression and Improves Prognosis in Human Bladder Cancer. J. Cel Mol Med 23 (3), 2064–2076. 10.1111/jcmm.14114 PMC6378218 30592142

[B39] Qiu Y. Qiu S. Deng L. Nie L. Gong L. Liao X. (2020). Biomaterial 3D Collagen I Gel Culture Model: A Novel Approach to Investigate Tumorigenesis and Dormancy of Bladder Cancer Cells Induced by Tumor Microenvironment. Biomaterials 256, 120217. 10.1016/j.biomaterials.2020.120217 32736172

[B40] Recalcati S. Locati M. Marini A. Santambrogio P. Zaninotto F. De Pizzol M. (2010). Differential Regulation of Iron Homeostasis during Human Macrophage Polarized Activation. Eur. J. Immunol. 40 (3), 824–835. 10.1002/eji.200939889 20039303

[B41] Rosenberg E. Baniel J. Spector Y. Faerman A. Meiri E. Aharonov R. (2013). Predicting Progression of Bladder Urothelial Carcinoma Using microRNA Expression. BJU Int. 112 (7), a–n. 10.1111/j.1464-410X.2012.11748.x 23387295

[B42] Rouanne M. Roumiguié M. Houédé N. Masson-Lecomte A. Colin P. Pignot G. (2018). Development of Immunotherapy in Bladder Cancer: Present and Future on Targeting PD(L)1 and CTLA-4 Pathways. World J. Urol. 36 (11), 1727–1740. 10.1007/s00345-018-2332-5 29855698

[B43] Siegel R. L. Miller K. D. Jemal A. (2020). Cancer Statistics, 2020. CA A. Cancer J. Clin. 70 (1), 7–30. 10.3322/caac.21590 31912902

[B44] Stevens R. G. Graubard B. I. Micozzi M. S. Neriishi K. Blumberg B. S. (1994). Moderate Elevation of Body Iron Level and Increased Risk of Cancer Occurrence and Death. Int. J. Cancer 56 (3), 364–369. 10.1002/ijc.2910560312 8314323

[B45] Sun Y. Liu W.-Z. Liu T. Feng X. Yang N. Zhou H.-F. (2015). Signaling Pathway of MAPK/ERK in Cell Proliferation, Differentiation, Migration, Senescence and Apoptosis. J. Receptors Signal Transduction 35 (6), 600–604. 10.3109/10799893.2015.1030412 26096166

[B46] Thévenod F. (2018). 15. Iron and its Role in Cancer Defense: A Double-Edged Sword. Met. Ions Life Sci. 18, 437–468. 10.1515/9783110470734-021 29394034

[B47] Torti S. V. Manz D. H. Paul B. T. Blanchette-Farra N. Torti F. M. (2018). Iron and Cancer. Annu. Rev. Nutr. 38, 97–125. 10.1146/annurev-nutr-082117-051732 30130469 PMC8118195

[B48] Torti S. V. Torti F. M. (2013). Iron and Cancer: More Ore to Be Mined. Nat. Rev. Cancer 13 (5), 342–355. 10.1038/nrc3495 23594855 PMC4036554

[B49] Walker C. Mojares E. del Río Hernández A. (2018). Role of Extracellular Matrix in Development and Cancer Progression. Int. J. Mol. Sci. 19 (10), 3028. 10.3390/ijms19103028 PMC6213383 30287763

[B50] Wang Y. Yu L. Ding J. Chen Y. (2018). Iron Metabolism in Cancer. Int. J. Mol. Sci. 20 (1), 95. 10.3390/ijms20010095 PMC6337236 30591630

[B51] Wang W. Green M. Choi J. E. Gijón M. Kennedy P. D. Johnson J. K. (2019). CD8+ T Cells Regulate Tumour Ferroptosis during Cancer Immunotherapy. Nature 569 (7755), 270–274. 10.1038/s41586-019-1170-y 31043744 PMC6533917

[B52] Wen H. Feng C.-c. Ding G.-x. Meng D.-l. Ding Q. Fang Z.-j. (2013). Med19 Promotes Bone Metastasis and Invasiveness of Bladder Urothelial Carcinoma via Bone Morphogenetic Protein 2. Ann. Diagn. Pathol. 17 (3), 259–264. 10.1016/j.anndiagpath.2012.11.004 23276457

[B53] Witjes J. A. Bruins H. M. Cathomas R. Compérat E. M. Cowan N. C. Gakis G. (2021). European Association of Urology Guidelines on Muscle-Invasive and Metastatic Bladder Cancer: Summary of the 2020 Guidelines. Eur. Urol. 79 (1), 82–104. 10.1016/j.eururo.2020.03.055 32360052

[B54] Wu T. Sempos C. T. Freudenheim J. L. Muti P. Smit E. (2004). Serum Iron, Copper and Zinc Concentrations and Risk of Cancer Mortality in US Adults. Ann. Epidemiol. 14 (3), 195–201. 10.1016/s1047-2797(03)00119-4 15036223

[B55] Xu T. Ding W. Ji X. Ao X. Liu Y. Yu W. (2019). Molecular Mechanisms of Ferroptosis and its Role in Cancer Therapy. J. Cel Mol Med 23 (8), 4900–4912. 10.1111/jcmm.14511 PMC6653007 31232522

[B56] Yamada Y. Kato M. Arai T. Sanada H. Uchida A. Misono S. (2019). Aberrantly Expressed PLOD 1 Promotes Cancer Aggressiveness in Bladder Cancer: a Potential Prognostic Marker and Therapeutic Target. Mol. Oncol. 13 (9), 1898–1912. 10.1002/1878-0261.12532 31199049 PMC6717764

[B57] Yang H. D. Eun J. W. Lee K.-B. Shen Q. Kim H. S. Kim S. Y. (2018). T-cell Immune Regulator 1 Enhances Metastasis in Hepatocellular Carcinoma. Exp. Mol. Med. 50 (1), e420. 10.1038/emm.2017.166 29303507 PMC5992982

[B58] Yi J. Zhu J. Wu J. Thompson C. B. Jiang X. (2020). Oncogenic Activation of PI3K-AKT-mTOR Signaling Suppresses Ferroptosis via SREBP-Mediated Lipogenesis. Proc. Natl. Acad. Sci. USA 117 (49), 31189–31197. 10.1073/pnas.2017152117 33229547 PMC7733797

[B59] Yin M. Joshi M. Meijer R. P. Glantz M. Holder S. Harvey H. A. (2016). Neoadjuvant Chemotherapy for Muscle-Invasive Bladder Cancer: A Systematic Review and Two-step Meta-Analysis. Oncologist 21 (6), 708–715. 10.1634/theoncologist.2015-0440 27053504 PMC4912364

[B60] Yuen H.-F. McCrudden C. M. Grills C. Zhang S.-D. Huang Y.-H. Chan K.-K. (2012). Combinatorial Use of Bone Morphogenetic Protein 6, Noggin and SOST Significantly Predicts Cancer Progression. Cancer Sci. 103 (6), 1145–1154. 10.1111/j.1349-7006.2012.02252.x 22364398 PMC7685053

[B61] Zhou D. Tang W. Xu Y. Xu Y. Xu B. Fu S. (2021). METTL3/YTHDF2 m6A axis Accelerates Colorectal Carcinogenesis through Epigenetically Suppressing YPEL5. Mol. Oncol. 15 (8), 2172–2184. 10.1002/1878-0261.12898 33411363 PMC8333777

[B62] Zhu S. Zhou H.-Y. Deng S.-C. Deng S.-J. He C. Li X. (2017). ASIC1 and ASIC3 Contribute to Acidity-Induced EMT of Pancreatic Cancer through Activating Ca2+/RhoA Pathway. Cell Death Dis 8 (5), e2806. 10.1038/cddis.2017.189 28518134 PMC5520710

